# Patterns of Symptom Tracking by Caregivers and Patients With Dementia and Mild Cognitive Impairment: Cross-sectional Study

**DOI:** 10.2196/29219

**Published:** 2022-01-27

**Authors:** Taylor Dunn, Susan E Howlett, Sanja Stanojevic, Aaqib Shehzad, Justin Stanley, Kenneth Rockwood

**Affiliations:** 1 Ardea Outcomes Halifax, NS Canada; 2 Department of Pharmacology Dalhousie University Halifax, NS Canada; 3 Division of Geriatric Medicine Dalhousie University Halifax, NS Canada; 4 Department of Community Health and Epidemiology Dalhousie University Halifax, NS Canada; 5 Geriatric Medicine Research Unit Nova Scotia Health Authority Halifax, NS Canada

**Keywords:** dementia, mild cognitive impairment, real-world evidence, patient-centric outcomes, machine learning, dementia stage, Alzheimer disease, symptom tracking

## Abstract

**Background:**

Individuals with dementia and mild cognitive impairment (MCI) experience a wide variety of symptoms and challenges that trouble them. To address this heterogeneity, numerous standardized tests are used for diagnosis and prognosis. myGoalNav Dementia is a web-based tool that allows individuals with impairments and their caregivers to identify and track outcomes of greatest importance to them, which may be a less arbitrary and more sensitive way of capturing meaningful change.

**Objective:**

We aim to explore the most frequent and important symptoms and challenges reported by caregivers and people with dementia and MCI and how this varies according to disease severity.

**Methods:**

This cross-sectional study involved 3909 web-based myGoalNav users (mostly caregivers of people with dementia or MCI) who completed symptom profiles between 2006 and 2019. To make a symptom profile, users selected their most personally meaningful or troublesome dementia-related symptoms to track over time. Users were also asked to rank their chosen symptoms from least to most important, which we called the symptom potency. As the stage of disease for these web-based users was unknown, we applied a supervised staging algorithm, previously trained on clinician-derived data, to classify each profile into 1 of 4 stages: MCI and mild, moderate, and severe dementia. Across these stages, we compared symptom tracking frequency, symptom potency, and the relationship between frequency and potency.

**Results:**

Applying the staging algorithm to the 3909 user profiles resulted in 917 (23.46%) MCI, 1596 (40.83%) mild dementia, 514 (13.15%) moderate dementia, and 882 (22.56%) severe dementia profiles. We found that the most frequent symptoms in MCI and mild dementia profiles were similar and comprised early hallmarks of dementia (eg, recent memory and language difficulty). As the stage increased to moderate and severe, the most frequent symptoms were characteristic of loss of independent function (eg, incontinence) and behavioral problems (eg, aggression). The most potent symptoms were similar between stages and generally reflected disruptions in everyday life (eg, problems with hobbies or games, travel, and looking after grandchildren). Symptom frequency was negatively correlated with potency at all stages, and the strength of this relationship increased with increasing disease severity.

**Conclusions:**

Our results emphasize the importance of patient-centricity in MCI and dementia studies and illustrate the valuable real-world evidence that can be collected with digital tools. Here, the most frequent symptoms across the stages reflected our understanding of the typical disease progression. However, the symptoms that were ranked as most personally important by users were generally among the least frequently selected. Through individualization, patient-centered instruments such as myGoalNav can complement standardized measures by capturing these infrequent but potent outcomes.

## Introduction

### Background

It is proving difficult to understand what constitutes successfully treated late-life dementia. This reflects in part the evolving understanding of Alzheimer disease (AD). Contemporary thinking sees AD as a biological construct, defined by biomarkers that can be detected in vivo or during autopsy [1]. In this formulation, AD is distinguished from Alzheimer dementia, a clinical syndrome [2]. However, this separation is not clear. In contrast to the prior view that a definitive diagnosis of AD could only be made at autopsy [3], it is now recognized that many people who meet the neuropathological criteria for AD do not have dementia when alive [4]. Few people with late-life dementia have *pure* AD; in the great majority, it is present along with many other neuropathological features [5,6]. Furthermore, even a full suite of neuropathological markers cannot distinguish between people who had dementia when alive and those who did not; other factors such as history of delirium [7], prior hospitalization [8], and degree of frailty are each important [9]. Similarly, a range of factors, from the level of education [10] to stimulating psychosocial and lifestyle experiences [11], is seen as potentially protective, even if less well-studied. A further challenge to defining successful treatment is that standard outcome measures, notably including the commonly used AD Assessment Scale–Cognitive subscale (ADAS-Cog), can underestimate meaningful clinical changes [12,13].

The new consensus on defining AD and the broader understanding of what gives rise to late-life dementia together have propelled a rethinking of which outcomes to measure in dementia and predementia clinical trials [14,15]. The Food and Drug Administration guidelines in 2018 [16] suggested that a single primary end point, which assesses both cognitive and functional effects (eg, the Clinical Dementia Rating [CDR] scale–Sum of Boxes [17,18]) may be used to evaluate treatment in early-stage patients with biomarker-defined AD. With this reevaluation, it may be useful to consider patient-reported impacts of treatment. This could be a less arbitrary means of understanding treatment efficacy compared with changes in biomarkers that tend to correlate poorly with clinical measures [19-24]. Along these lines, the lack of correlation between clinical manifestations of the disease and biomarker positivity has motivated the reconsideration of a purely biological definition of AD, suggesting that the disease designation be restricted to people who combine biomarker positivity with specific AD phenotypes [25].

Patient-centric outcome measures, in which patient (and caregiver) preferences are directly incorporated and measured, have slowly gained traction in clinical trials and research communities [26], including an endorsement from the Food and Drug Administration [27]. By giving a voice to the patient, we can achieve more meaningful and interpretable measures of treatment benefit [28], as seen in some dementia research, including clinical trials of people with AD receiving donepezil [29] and galantamine [30], which used goal attainment scaling [31,32] as a primary outcome. Here, personalized outcomes offered highly sensitive measures of change that were viewed as clinically meaningful by patients and caregivers [13]. This approach also provided additional insights into what is most important to this population, for example, an unanticipated treatment benefit in the troubling symptom of verbal repetition [33,34]. Indeed, from mild cognitive impairment (MCI), which is the symptomatic predementia stage of AD [35], to the severe stage of dementia, patients are troubled by diverse sets of cognitive, functional, and behavioral symptoms. However, we lack a comprehensive inventory of symptoms across the disease spectrum and their susceptibility to treatment. Surveys of symptoms are few, in part as they are expensive. For this purpose, the web-based environment can be well-suited.

Our group has shown that data on people living with MCI and dementia and their caregivers can be acquired with an internet-based tool called myGoalNav Dementia (previously SymptomGuide Dementia; developed by Ardea Outcomes). This symptom tracking platform provides a large library from which users can identify and track dementia symptoms that are most important to them [36]. Note the distinction between a symptom being present—as in a *tick box* survey of symptom prevalence—and one being important to individual patients and caregivers. Earlier, we had used myGoalNav to investigate construct validity with the Dependence Scale [37]; identify clusters of neuropsychiatric symptoms [38]; characterize the symptoms of verbal repetition [39], misplacing objects [40], and agitation [41]; and evaluate donepezil in a 6-month open-label study [42]. Here, we use myGoalNav Dementia to better understand the patterns of dementia symptom tracking with the severity of impairment, as staged by a machine learning algorithm.

### Objective

The aims of this cross-sectional study are three-fold: (1) to compare symptom frequency by stage, (2) to compare symptom importance by stage, and (3) to examine the relationship between frequency and importance. In doing so, our overall goal is to demonstrate the usefulness and the types of insights gained from web-based symptom tracking in people with dementia and MCI.

## Methods

### Data Collection

The data are from the myGoalNav Dementia platform, previously called SymptomGuide Dementia. Launched as a website in 2007 for people with cognitive impairment and their caregivers, the key feature of the platform is a library (or *menu*) of common dementia-related symptoms and challenges. The library was developed over many years, beginning with a qualitative analysis of personalized treatment goals set by patients, caregivers, and clinicians in 2 clinical trials of anticholinesterase inhibitors [29,30] and from a memory clinic in Halifax, Nova Scotia. From this qualitative analysis, an expert geriatrician panel reviewed the first draft using the Delphi method [43] and arrived at a library of 60 symptoms [37,41]. In 2018, myGoalNav Dementia was redesigned as an iOS and Android mobile app, and based largely on user feedback, the library was expanded to 67 symptoms, each with 2 to 12 (median 9) plain language descriptors that provide an additional level of detail into symptom manifestation.

In addition to providing users with educational information and management tips for each symptom, users have the option of choosing from the library any number of symptoms and relevant descriptors to track over time, which are important to them or the person for whom they care. If they wish for further personalization, users can log *other* symptoms and descriptors that do not appear in the library. The set of initial symptoms selected by a user is called their baseline symptom profile, which they may supplement with additional demographic information such as age, gender, and living arrangements. As an optional step, users are also asked to rank their chosen symptoms from most to least important or troublesome.

myGoalNav Dementia is currently being retooled as a mobile-first web-based care app that better facilitates shared decision-making and improves the quantity and quality of touchpoints between the provider and patient. The transition to web-based technologies affords us flexibility in incorporating our dementia staging model within the app in the future. Currently, it is slated to undergo pilot testing with the collaboration of our health care provider partners and is no longer available as a community app.

myGoalNav was not designed to be an inventory of every dementia-related problem that an individual might experience. Rather, the library facilitates the selection of those symptoms that are most meaningful to each participant. For this analysis, we excluded outlier profiles created by individuals who chose >22 symptoms (95th percentile).

### Staging Dementia

In 2013, we developed an artificial neural network model to stage dementia, which was trained on data from 320 memory clinic patients [42]. That model was updated in 2020 using a support vector machine–supervised learning algorithm trained on 717 patients [44]. Data from these patients were captured with myGoalNav in a memory clinic, a long-term care study [45], and a dementia clinical trial [42]. Patients were staged by a clinician using either the Functional Assessment Staging Test or the Global Deterioration Scale into 1 of 4 stages: MCI or mild, moderate, or severe dementia. Patient age and their symptom profiles served as inputs to train the model to predict the dementia stage. Further details of the model, including algorithm choice and performance, can be found in the study by Shehzad et al [44].

### Statistical Analysis

User characteristics and demographics were summarized, and the differences between stages were tested. Categorical variables were summarized as percentages of users and tested using the Pearson chi-square test. Continuous variables were summarized as means and SDs or medians (lower and upper quartiles) and tested using the Kruskal–Wallis *H* test.

To compare symptom frequency by disease stage (objective 1), we first fit a logistic regression model with the number of profiles selecting each symptom in each stage as the dependent variable. Symptom name, stage, and the interaction between the 2 were included as independent variables. The estimates from this model were transformed to stage-specific symptom frequencies with 95% CIs.

We investigated the frequency differences between the stages in 2 ways. First, we computed Pearson correlation coefficient *r* on frequencies between each pair of stages, where a higher *r* coefficient indicates greater similarity in symptom selection. Second, we quantified the degree to which a symptom was associated with increasing or decreasing disease severity. This was accomplished by modifying our logistic regression model so that the stage is treated as a monotonic predictor variable [46] rather than a categorical variable without ordering. The estimates from this model can be interpreted as the average difference in frequency (on the log-odds scale) between adjacent stages (*Supplementary Methods* section in Multimedia Appendix 1 [46-49]).

To compare symptom importance by stage (objective 2), we began by defining *relative* symptom importance within a symptom profile as the weighted rank or *potency*:


*w_ij_=r_ij_/n_j_*
**(1)**

where *n_j_* is the number of symptoms in profile *j,* and *r_ij_* is the rank (out of *n_j_*) given to symptom *I*. Higher potency *w_ij_* corresponds to the higher relative importance of symptom *i* to the user of profile *j*.

Next, to model this proportion while accounting for the wide range in the number of tracked symptoms among myGoalNav users, we used logistic regression with *w_ij_* as the dependent variable; *n_j_* as case weights; and categorical independent variables of symptom name, stage, and their interaction. As with the frequency analysis, we estimated pairwise similarity in stages by computing Pearson correlation coefficients between potency estimates.

To investigate the relationship between symptom frequency and potency (objective 3), we visualized the relationship by plotting frequency against potency estimates. We quantified the strength of these relationships using Pearson correlation.

No missing data were imputed for this study. As mentioned, we removed outlier profiles with >22 symptoms (95th percentile) but otherwise did not exclude users for missing or abnormal data. All analyses were performed in R version 4.0.2 (R Foundation for Statistical Computing) [50] using tidyverse packages [51].

### Ethics

Clinic data were collected after having obtained written informed consent. Participants completed a form that allowed their anonymized data to be analyzed for research purposes. Data collection was approved by the research ethics committee of the Nova Scotia Health Authority. myGoalNav users consented to terms of use, which included allowing their data to be aggregated and used for research purposes. Users were assured that the research findings would be presented in a manner that would not disclose personally identifying information.

### Data Availability

Aggregated data are presented in Table S1 (symptom frequency) and Table S2 (symptom potency) of Multimedia Appendix 1. For confidentiality reasons, user-level data cannot be made publicly available. Access to deidentified data may be provided upon reasonable request.

## Results

### The Sample

To date, 12,347 users have signed up for myGoalNav, and of these, 4213 (34.12%) users created a symptom profile. Of the 4213 users, after removing the profiles tracking >22 symptoms, our final sample size was 3909 (92.78%) profiles, with creation dates ranging from May 15, 2006, to November 15, 2018.

The great majority, 96.01% (3753/3909), of these symptom profiles were made on the web platform, with 3.99% (156/3909) from the later mobile app. Most profiles (3792/3909, 97.01%) were completed by caregivers, and the remaining (117/3909, 2.99%) were completed by participants (people with cognitive impairment) on their own behalf. The staging algorithm led to the following distribution of severity across the 3909 profiles: 917 (23.46%) MCI, 1596 (40.82%) mild dementia, 514 (13.15%) moderate dementia, and 882 (22.56%) severe dementia.

Participant characteristics and demographics are summarized by stage in Table 1. With increasing severity from MCI to moderate dementia, participants tended to be older, less well-educated, more likely to identify as women, and less likely to live on their own. A minority of caregivers (1361/3792, 35.89% users) also provided information about themselves. Most caregivers were women (515/802, 64.2%), aged 46-55 years (184/583, 31.6%), and spouses or partners of the participant (156/524, 29.8%).

**Table 1:** Baseline characteristics of the myGoalNav participants, stratified by stage (N=3909).

Characteristic^a^			Total		MCI^b^ (n=917)		Mild (n=1596)		Moderate (n=514)		Severe (n=882)		Test statistic^c^							
													*P* value			*H* test (*df*)			Chi-square (*df*)	
Age (years; n=2473), mean (SD)			75.4 (12.4)		70.5 (13.2)		74.5 (12.1)		80.9 (8.7)		78.2 (11.9)		<.001			192.8 (3)			N/A^d^	
**Gender** **(n=3909), n (%)**														.046			N/A			8.0 (3)
	Man	976 (37.7)^e^		228 (40.9)^f^		431 (39)^g^		127 (33.7)^h^		190 (34.7)^i^										
	Woman	1611 (62.3)^e^		329 (59.1)^f^		674 (61)^g^		250 (66.3)^h^		358 (65.3)^i^										
Number of symptoms (n=2587), median (Q1-Q3)			4 (2-7)		2 (1-4)		5 (3-8)		7 (4-11)		4 (2-7)		<.001			669.7 (3)			N/A	
**Education (n=1337), n (%)**														.18			N/A			12.7 (9)
	Secondary school or less	625 (46.7)		117 (40.8)^j^		283 (45.7)^k^		99 (52.1)^l^		126 (52.3)^m^										
	Trade school	71 (5.3)		14 (4.9)^j^		32 (5.2)^k^		9 (4.7)^l^		16 (6.6)^m^										
	Undergraduate	439 (32.8)		108 (37.6)^j^		211 (34.1)^k^		53 (27.9)^l^		67 (27.8)^m^										
	Graduate	202 (15.1)		48 (16.7)^j^		93 (15)^k^		29 (15.3)^l^		32 (13.3)^m^										
**Living arrangement (n=2013), n (%)**														<.001			N/A			126.5 (12)
	Alone	290 (14.4)		67 (14.9)^n^		143 (15.8)^o^		35 (12.2)^p^		45 (12.1)^q^										
	Assisted living	315 (15.6)		39 (8.7)^n^		102 (11.3)^o^		71 (24.8)^p^		103 (27.8)^q^										
	With caregiver	335 (16.6)		59 (13.1)^n^		153 (16.9)^o^		54 (18.9)^p^		69 (18.6)^q^										
	With family or friend	1038 (51.6)		283 (62.9)^n^		494 (54.5)^o^		120 (42)^p^		141 (38)^q^										
	With paid companion	35 (1.7)		2 (0.4)^n^		14 (1.5)^o^		6 (2.1)^p^		13 (3.5)^q^										

^a^N is the number of users with nonmissing values.

^b^MCI: mild cognitive impairment.

^c^Comparisons between stages: Pearson chi-square test and Kruskal–Wallis *H* test.

^d^N/A: not applicable.

^e^Sample size, n=2587.

^f^Sample size, n=557.

^g^Sample size, n=1105.

^h^Sample size, n=377.

^i^Sample size, n=548.

^j^Sample size, n=287.

^k^Sample size, n=619.

^l^Sample size, n=190.

^m^Sample size, n=241.

^n^Sample size, n=450.

^o^Sample size, n=906.

^p^Sample size, n=286.

^q^Sample size, n=371.

### Symptom Frequency

**Figure 1:**
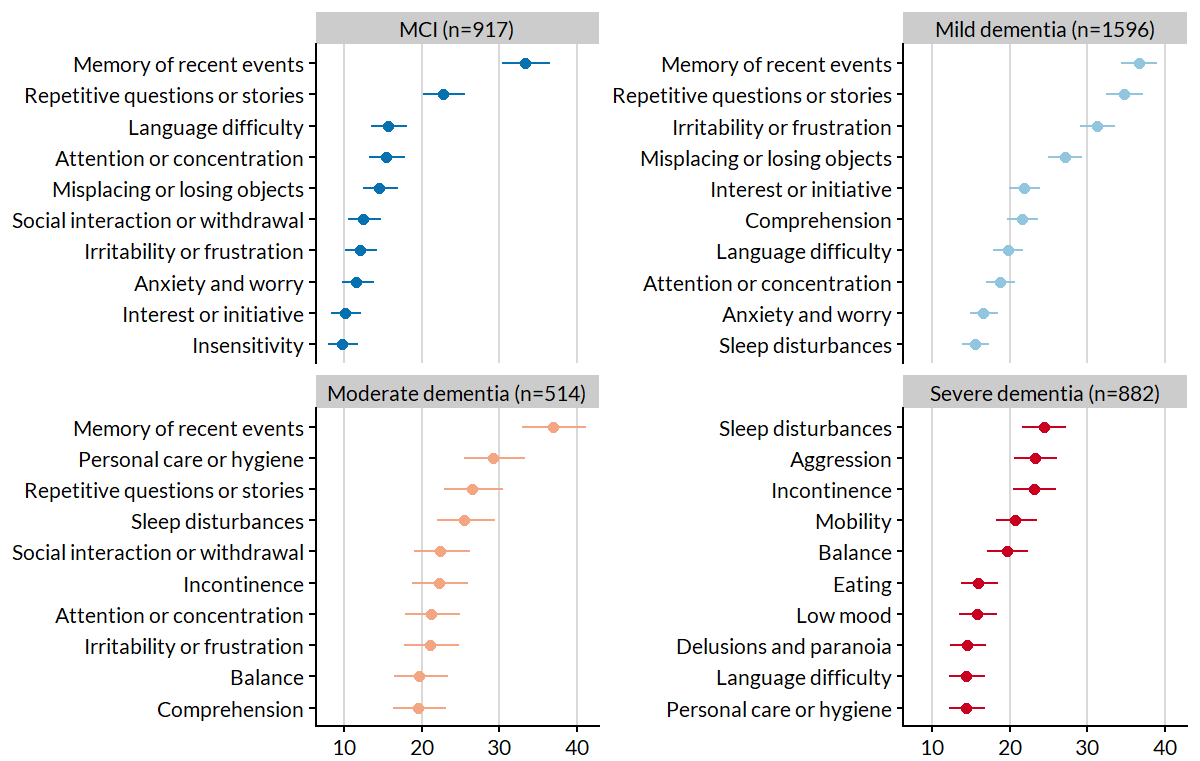
The 10 most frequent symptoms tracked by baseline myGoalNav profiles, stratified by stage. Data are presented as point estimates and 95% CIs from the logistic regression model. MCI: mild cognitive impairment.

Figure 1 depicts the 10 most frequent symptoms in each stage. In MCI, mild dementia, and moderate dementia profiles, the most common symptom was memory of recent events: 33.41% (1306/3909), 36.71% (1435/3909), and 36.99% (1446/3909) of profiles, respectively. This early hallmark of dementia was tracked much less often at the severe stage, where 9.79% (383/3909) of profiles showed it being tracked. Other symptoms were tracked more often with greater severity. For example, sleep disturbance tracking increased from 6.7% (61/917) in MCI profiles to 15.47% (247/1596) in mild dementia profiles and to 25.5% (131/514) in moderate dementia profiles and was the most frequently tracked symptom in the severe dementia profiles (215/882, 24.4%).

In addition to memory of recent events, MCI profiles were best characterized by repetitive questions or stories; no other symptom had a tracking frequency >20%. Those with mild and moderate dementia profiles showed more variety in symptom selection, with 6 and 8 symptoms >20% frequency, whereas those with severe dementia profiles were slightly more uniform, with 4 symptoms above that mark.

**Figure 2:**
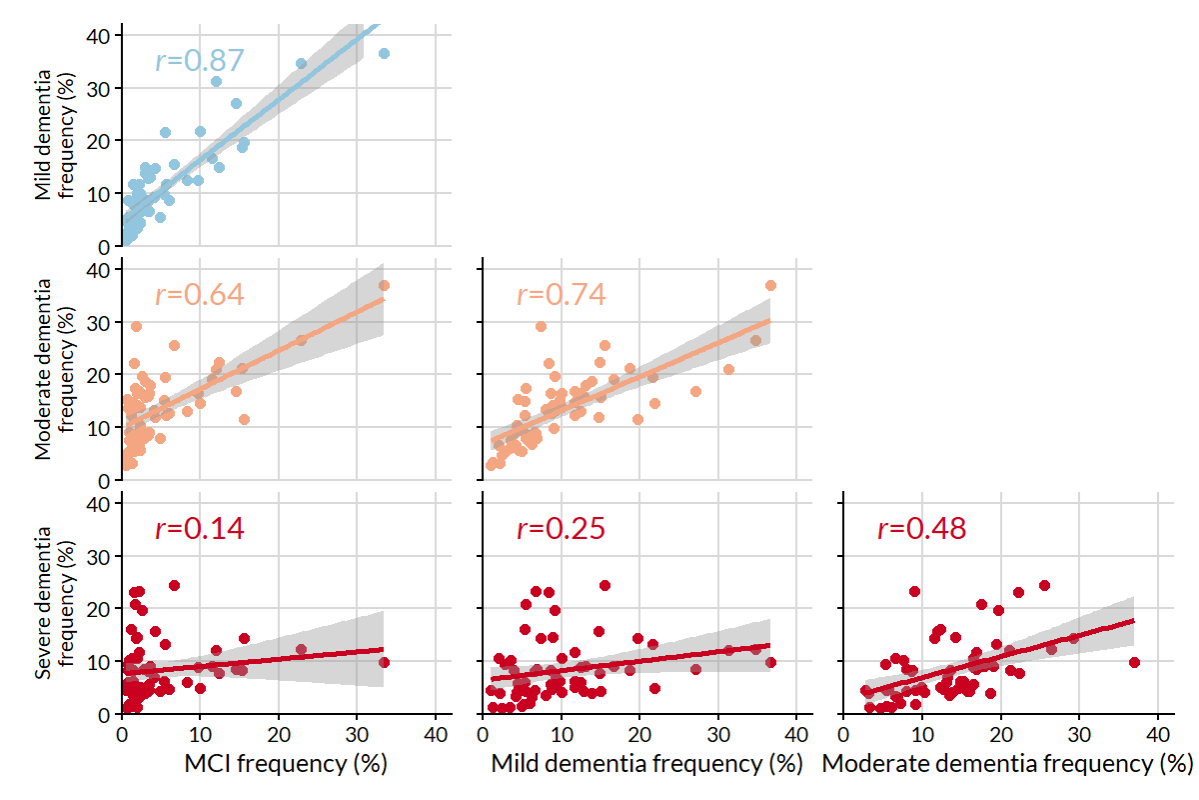
Pairwise relationships of symptom frequency between stages. Pearson correlation coefficients (*r*) and lines of best fit (with 95% CIs) are displayed for each pair. MCI: mild cognitive impairment.

How frequencies varied is also illustrated by changes in the correlations between pairs of symptoms drawn from adjacent stages (Figure 2). Using this metric, the most similar stages (ie, highest correlation coefficient) were MCI and mild dementia. Indeed, those with MCI and mild dementia profiles shared 80% (8/10) of their most frequent symptoms. To a lesser degree, individuals with moderate dementia profiles had symptom frequencies similar to MCI and mild dementia profiles. There were four symptoms shared among the top 10 of these three stages: attention or concentration, irritability or frustration, memory of recent events, and repetitive questions or stories. By a large margin, severe dementia profiles had the most distinct set of frequent symptoms, although the correlation increased with increasing severity: *r*=0.14, 0.25, and 0.48 in MCI, mild dementia, and moderate dementia profiles, respectively. The most frequent symptoms in severe dementia profiles were characteristic of loss of independent function (incontinence, mobility, eating, and personal care or hygiene) and more extreme behavioral problems (aggression, low mood, and delusions and paranoia).

**Figure 3:**
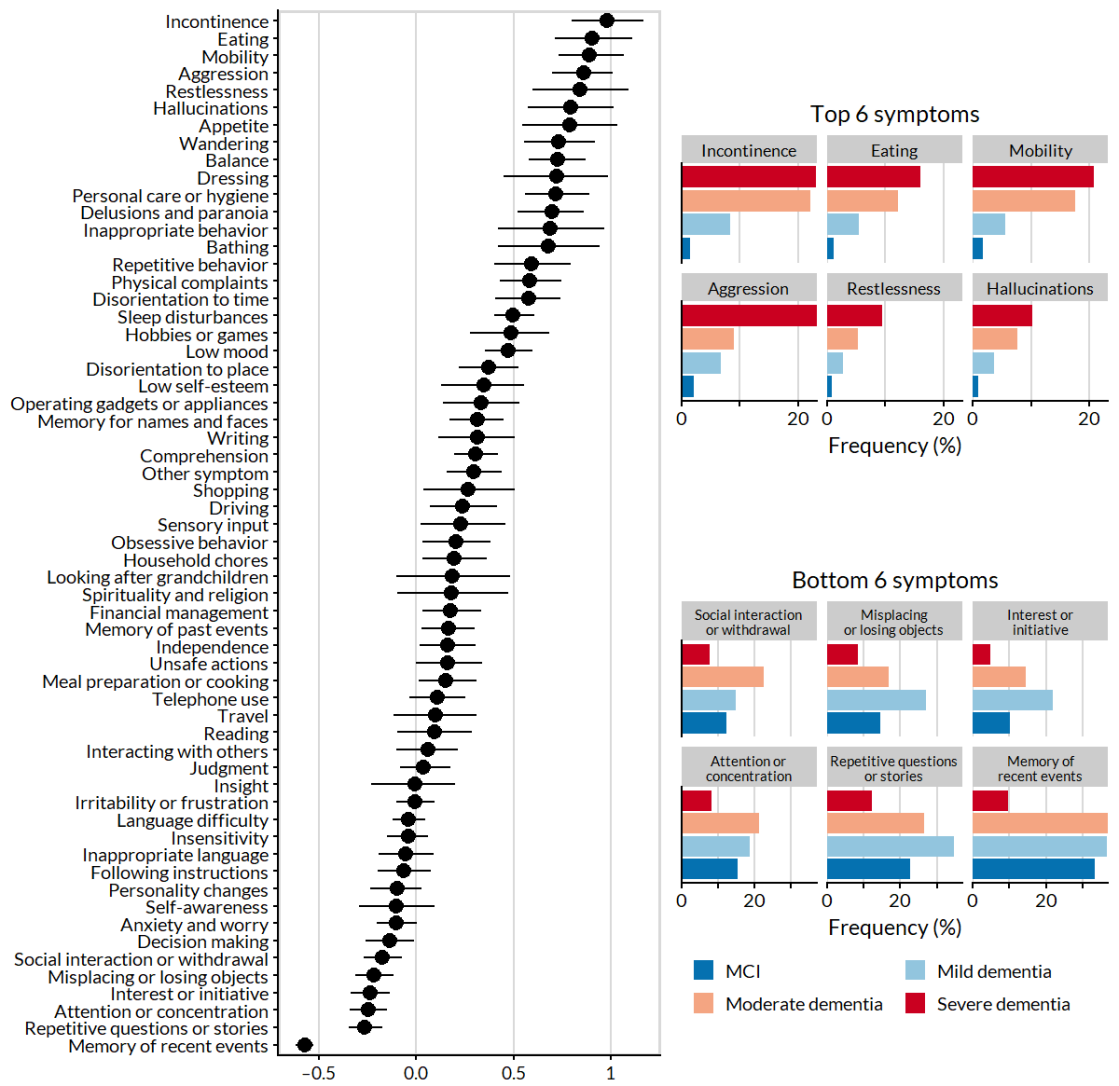
Estimated symptom monotonicity, where higher values indicate the increasing frequency with ordered stage. Data are presented as point estimates and 95% CIs from the logistic regression model, with stage as a monotonic predictor (left). Stage-specific frequencies for the 6 symptoms with the highest positive monotonicity and the 6 symptoms with the highest negative monotonicity (right). MCI: mild cognitive impairment.

Model-estimated monotonicity of frequency with dementia severity is shown for each symptom in Figure 3. More symptoms exhibited positive monotonicity (36 symptoms with lower 95% CI >0) than negative (7 symptoms with upper 95% CI <0). The 6 symptoms with the highest positive and negative monotonicity are shown on the right in Figure 3.

Overall frequency and frequency by stage for each symptom are summarized in Table S1 in Multimedia Appendix 1.

### Symptom Potency

**Figure 4:**
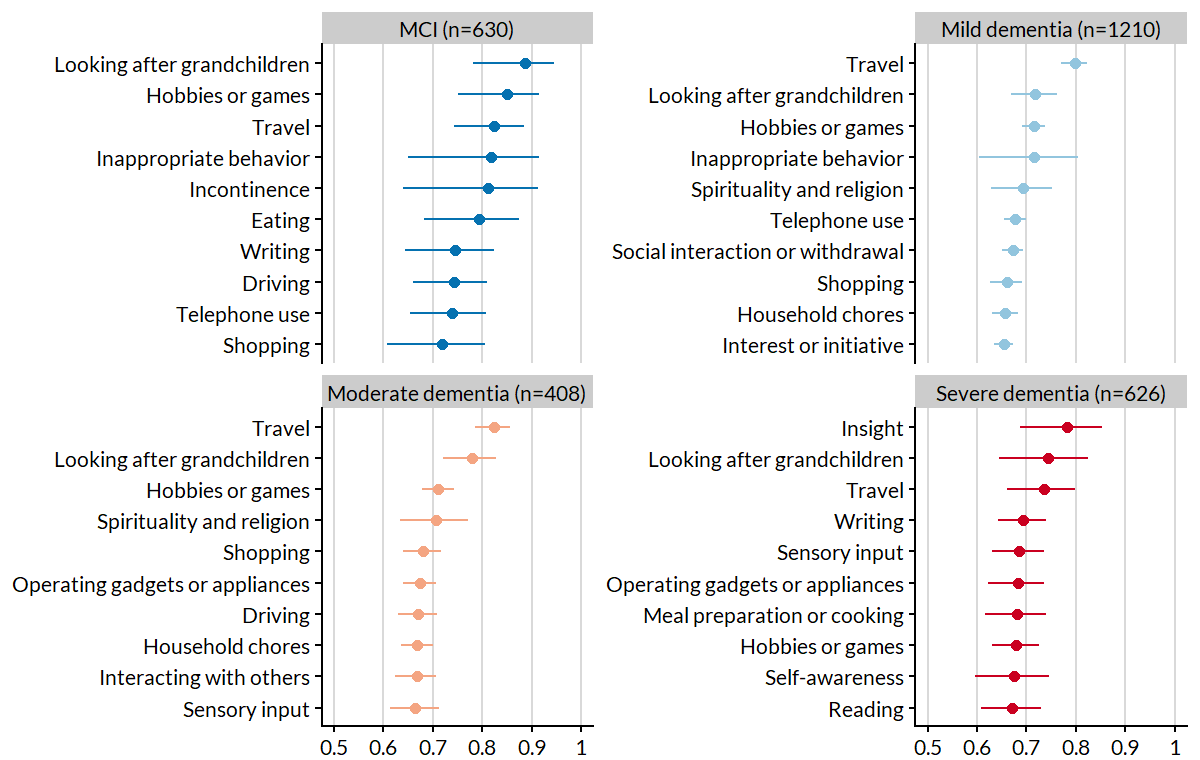
The 10 most potent symptoms tracked by baseline myGoalNav profiles, stratified by stage. Data are presented as point estimates and 95% CIs from the logistic regression model. MCI: mild cognitive impairment.

As the ranking of symptoms is not compulsory on myGoalNav, the potency analysis involved 2874 symptom profiles (632, 21.99% MCI, 1207, 41.99% mild dementia, 402, 13.98% moderate dementia, and 632, 21.99% severe dementia). The model estimates of the 10 most potent symptoms by stage are shown in Figure 4, and the rest can be found in Table S2 in Multimedia Appendix 1. Of the 2874 symptoms, 2 (0.07%) symptoms stood out as important regardless of stage—travel and looking after grandchildren—which were among the top 3 most potent symptoms in each stage.

**Figure 5:**
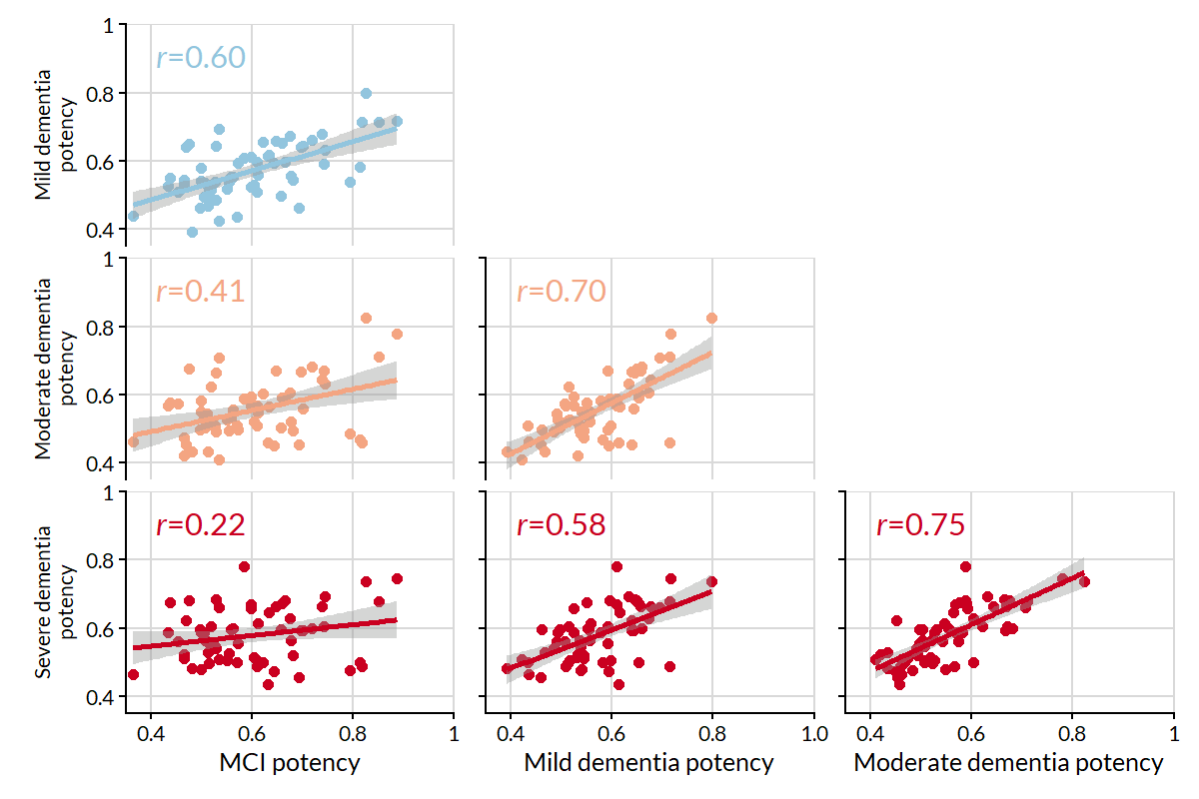
Pairwise relationships of symptom potency between stages. Pearson correlation coefficients (*r*) and lines of best fit (with 95% CIs) are displayed for each pair. MCI: mild cognitive impairment.

Figure 5 shows the pairwise relationship of symptom potency between stages. The most similar pairs of stages were moderate dementia profiles with mild and severe dementia profiles. The greatest differences in potency were MCI profiles with moderate and severe dementia profiles.

### Frequency and Potency

**Figure 6:**
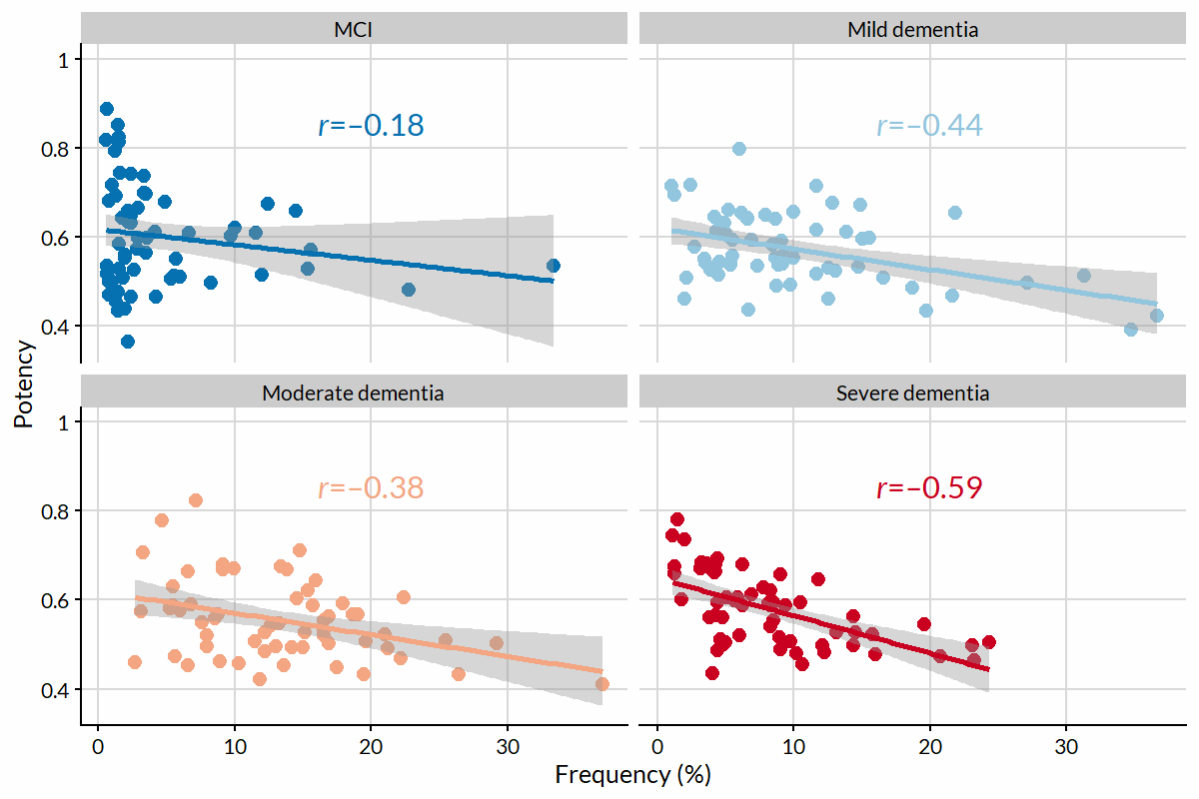
The relationship between symptom frequency and potency, stratified by stage. Pearson correlation coefficients (*r*) and lines of best fit (with 95% CIs) are displayed for each stage. MCI: mild cognitive impairment.

We discovered a clear discrepancy between symptoms that were most frequent (Figure 1) and those that were most potent (Figure 3). Only interest or initiative was both highly frequent (632/2874, 21.99%) and potent (potency 0.65, 95% CI 0.63-0.67) among the mild dementia symptom profiles. Symptom frequency was negatively correlated with potency regardless of severity (Figure 6). The degree of association varied by stage from weakly correlated in MCI profiles (*r*=−0.18) to moderately correlated in severe dementia profiles (*r*=−0.59). The patterns (or trajectories) of potency and frequency are visualized for selected symptoms in Figure S1 in Multimedia Appendix 1.

## Discussion

### Principal Findings

This study used a machine learning algorithm, which was trained with clinician-staged data, to investigate how symptom tracking in web-based profiles differed by the severity of cognitive impairment (MCI and dementia). Our key finding was the large distinction between what is common and what is most important to people with cognitive impairment and their caregivers. This reinforces the importance of capturing the patients’ and caregivers’ voices when determining clinically meaningful changes in MCI and dementia trials. The fact that we were able to discover associations that were sensible and meaningful suggests that information collected on the web has the potential to yield useful insights into this population. As clinical treatments that focus on single-protein abnormalities understandably exclude people who do not conform to classic profiles consistent with those abnormalities, there is a need for data on real-world experiences of the larger constituency of people in whom dementia reflects a variety of disease processes.

Symptom tracking frequency was similar across MCI and mild and moderate dementia profiles, with all pairwise correlations between frequencies *r*>0.5. Memory of recent events and repetitive questions or stories were notably among the top 3 symptoms for all 3 stages. However, neither of these symptoms appeared among the 10 most frequent symptoms in the severe dementia profiles, which differed appreciably from the other stages (all *r*<0.5). With severe impairment, the early hallmarks of dementia (such as impaired memory) are present but become secondary to more distressing symptoms. In particular, we found that increasing disease severity was most associated with loss of independent function (incontinence, eating, and mobility) and behavioral problems (aggression, restlessness, and hallucinations).

The most potent (relatively important) symptoms were generally those related to disruptions in everyday life that, although less common, had great meaning when they occurred, for example, problems in looking after grandchildren, hobbies or games, and travel. Declining cognition may be a concern; however, the resulting changes to routine can be especially distressing to affected people and their families. Potency was most similar between stages of similar severity. For instance, the strongest relationships were between the adjacent pairs of MCI–mild dementia, mild-to-moderate dementia, and moderate-to-severe dementia, with all *r*≥0.6.

As the large majority of our users (3792/3909, 97.01%) are caregivers, we see it reasonable to conceptualize symptom potency as an indicator of caregiver burden—the more distressing and burdensome symptoms are more likely to be ranked as highly potent by our users. In this context, we see in this study that impairment in instrumental activities of daily living (IADL; higher-order functions such as travel, meal preparation or cooking, shopping, and telephone use) place the most burden on caregivers, relative to basic activities of daily living and cognitive and behavioral symptoms. Our results are aligned with those of previous studies investigating symptoms and caregiving difficulties across the stages of the disease. A survey conducted by Alzheimer Europe [52] asked 1181 caregivers of patients with mild, moderate, or severe dementia about their current and most distressing symptoms. Problems with activities of daily living (including financial activities, shopping, cooking, and telephone use) were the most prevalent (reported by 96% of caregivers) and most problematic (68% of caregivers) symptoms, followed by behavioral symptoms (50%). In a multicenter study of 328 informal caregivers of patients who mostly experienced mild or moderate AD, IADL deficits were associated with more caregiver burden, as measured by the Zarit Burden Interview [53]. A prospective cohort study of 135 patients, ranging from those with MCI (CDR=0.5) to those with severe (CDR=3) dementia, found that depressive state in caregivers was independent of cognitive decline but was strongly associated with a decline in IADLs and delusional behavior [54].

Across stages, symptom frequency was negatively correlated with potency, and the strength of this relationship generally increased with increasing severity. We believe this pattern reflects the nature of the disease course. Early on, and especially before diagnosis, gradual change in cognitive function will be both apparent and alarming to the person living with the problems and to their caregivers; this likely underlies why the typical symptoms are still fairly potent. As deterioration increases, so does the heterogeneity in its manifestation. The typical clinical presentation becomes the accepted norm (ie, still frequent but less potent), and the impact on quality of life becomes more potent.

The contrast in symptom frequency and potency also has important implications for the measurement and interpretation of clinically meaningful changes. Outcome measures must be practical to use in that they do not overburden the informant with long interview times [55]. Including several items also has the risk of probing irrelevant information, which can affect an instrument’s sensitivity to change. Striking a balance between robustness and concision is a substantial challenge in outcomes development, especially in dementia, where patient priorities are highly variable, as shown in this study. A number of best practices [56] and statistical techniques [15] exist to tackle this heterogeneity using standardized outcome measures; however, we believe that an individualized approach guided by the patient and caregiver’s priorities for treatment is a simpler solution to a complex problem. By focusing on what matters most, we can guarantee that any changes measured are meaningful.

Although there are too many symptoms to compare with the literature, we draw attention to a few, such as the following: repetitive questions or stories, sleep disturbance, and interest or initiative. The stage-specific frequencies and potencies of these symptoms are visualized in Figure S1 in Multimedia Appendix 1.

We have explored the symptom of verbal repetition (here, repetitive questions or stories) in the Atlantic Canada AD Investigation of Expectations trial (open-label trial of donepezil in mild-to-moderate AD) [33] and the Video-Imaging Synthesis of Treating AD trial (randomized controlled trial of galantamine in mild-to-moderate AD) [34]. In both trials, where goal attainment scaling was the primary outcome, reduction of verbal repetition was identified as a goal of treatment in 46% and 44% of patients, respectively. This symptom notably improved more often in patients treated with galantamine than in those treated with a placebo. Our data were consistent with these secondary analyses and with a 2013 analysis of myGoalNav users [39], where verbal repetition was commonly tracked (26% overall), especially in the mild stage (35%). Hwang et al [57] also found verbal repetition to be an early sign of dementia that was troublesome to caregivers. When patients and caregivers are allowed a voice, we see that verbal repetition is important, common, and responsive, although it typically goes unmeasured by standard tests.

We found the increasing frequency of sleep disturbances with severity to be compelling, especially as it was the most commonly tracked symptom in severe dementia profiles. Similarly, Moe et al [58] found sleep disturbances to increase with disease severity in a sample of 78 AD patients. Sleep disturbances are also important at earlier stages; its prevalence in patients with MCI was estimated to be 14% to 59% in a review of 15 studies [59]. Here, the frequency was 7% in myGoalNav MCI profiles, which is unsurprisingly lower than prevalence estimates but still ranked as the 12th most frequent MCI symptom among 60 symptoms. Growing evidence suggests that sleep disturbances are a risk factor for AD [60,61], which underlines the importance of further study during predementia stages.

The symptom of interest or initiative describes a patient who is losing interest in everyday life and who has become disengaged from others and the world around them. It is common, distressing for caregivers [62], and a potential risk factor for progression from MCI to dementia [63]. In the Video-Imaging Synthesis of Treating AD trial, decreased initiation was a treatment goal for 71 of the 84 participants with mild-to-moderate AD (out of 130) who were described as having the symptom [64]. Unsurprisingly, it emerged as a noteworthy symptom in our analyses, particularly in mild dementia profiles, where it was among both the 10 most frequent and most potent symptoms. This symptom may also be important for its sensitivity to change. In a survey of caregiver and patient judgment on changes in symptoms, apathy was the neuropsychiatric symptom that improved the most in patients with MCI and AD treated with the nutritional intervention Fortasyn Connect [65].

With no approved treatment for individuals with prodromal AD, some promise is seen in nonpharmacological interventions, especially those that combine multiple lifestyle modifications such as diet and exercise [66,67]. The Finnish Geriatric Intervention Study to Prevent Cognitive Impairment and Disability study showed that a multimodal intervention combining diet, exercise, cognitive training, and vascular risk monitoring might improve or maintain cognitive functioning among older individuals who are at risk of dementia [68]. Nutritional interventions are also being developed to tackle dietary deficiencies associated with AD pathology. A medical food (Fortasyn Connect) has been shown to improve memory in randomized controlled trials of patients with mild [69,70], but not mild-to-moderate [71], AD over 3 and 6 months of treatment. These trials were followed by the 24-month LipiDiDiet randomized controlled trial for individuals with prodromal AD [72]. There was no significant treatment effect on the neuropsychological test battery primary end point; however, there was evidence of cognitive and functional benefit, as assessed by the secondary CDR–Sum of Boxes end point, and this effect increased with better baseline cognition. In addition, results of the LipiDiDiet 36-month extension trial showed significant treatment effects on multiple measures of cognition, function, and disease progression [73]. Taken together, these studies highlight the potential for early interventions in dementia, notably with lifestyle modification, especially dietary lifestyle. With this comes a need for adequately sensitive outcomes to detect meaningful effects at early stages, for which individualized symptom tracking may be a solution [13].

### Limitations

Our data must be interpreted with caution as it comprises observer-reported tracking data completed mostly by caregivers of people with dementia, who were not supervised in how they described or recorded the symptoms of the people for whom they were caring. There may have been a selection bias toward caregivers with higher functioning who can more easily locate and operate myGoalNav. Furthermore, as the myGoalNav is not a checklist of symptoms, the tracking frequencies presented here are distinct from symptom *prevalence*. There are also limitations in the development of the staging algorithm model, such as potential bias in the training data because of clinician facilitation [44].

### Conclusions

Our results emphasize the importance of patient-centricity in evaluating interventions for MCI and dementia [74-76]. A personalized outcome, for example, of a grandparent being able to travel and look after their grandchild independently, will be more meaningful to the patient and their family compared with a 4-point change on the ADAS-Cog, which is considered as the main criterion for benefit. Asking patients about what is most important is sensitive to change and is inherently clinically meaningful [36,77,78]. This can be especially valuable in the predementia stages, where standard outcomes such as the ADAS-Cog and Mini Mental State Examination lack sensitivity [79,80]. Tools such as myGoalNav Dementia, and individualized outcome measures such as goal attainment scaling [81,82], are pragmatic ways of capturing the patient voice in real-world and clinical trial settings.

## Appendix

Multimedia Appendix 1 Supplementary methods detailing the logistic regression model of symptom frequency with dementia stage as a monotonic predictor. Table S1 contains all symptom frequencies by stage and Table S2 contains all symptom potencies by stage. Figure S1 depicts the patterns of potency and frequency for selected symptoms.

## Abbreviations

AD: Alzheimer disease
ADAS-Cog: Alzheimer Disease Assessment Scale–Cognitive subscale
CDR: Clinical Dementia Rating
IADL: instrumental activities of daily living
MCI: mild cognitive impairment
