# Supplementary material for: Patterns of Symptom Tracking by Caregivers and Patients With Dementia and Mild Cognitive Impairment: Cross-sectional Study
Source: J Med Internet Res. 2022 Jan 27;24(1):e29219. doi: 10.2196/29219 (PMC8832273; doi:10.2196/29219)
Supplement: Multimedia Appendix 1 [file jmir_v24i1e29219_app1.docx]

Multimedia Appendix

# Supplementary Methods

Our goal with this analysis is to estimate symptom-level trends in tracking frequency with dementia stage by modeling it as a monotonic predictor (consistently negative or positive across values) using the method proposed by Bürkner and Charpentier [46]. For each stage $j$, we consider the number of total profiles in that stage ($n_{j}$) in relation to the number of profiles tracking a symptom $i$ ($n_{ij}$). We start with a binomial sampling model for $n_{ij}$:

$$n_{ij}\sim\text{Binomial}(p_{ij},n_{j})$$

where $p_{ij}$ is the proportion of profiles in stage $j$ tracking symptom $i$ (the frequency). We model $p_{ij}$ conditional on symptom, stage and their interaction:

$$\text{logit}(p_{ij})=\alpha+\beta_{i}\text{symptom}_{i}+\gamma_{0} \text{mo}(\text{stage}_{j},\zeta_{0j})+\gamma_{i} \text{symptom}_{i} \text{mo}(\text{stage}_{j},\zeta_{ij})$$

where we have imposed the monotonicity constraint on the stage variable by using the monotonic transform:

$$\text{mo}(\text{stage}_{j},\zeta_{ij})=J\sum_{j=1}^{\text{stage}_{j}} \zeta_{ij}$$

where $J+1$ is the number of of stages (4) and $\zeta_{ij}$ are simplex parameters which satisfy $\zeta_{ij}\in[0,1]$ and $\sum_{j=1}^{\text{stage}_{j}} \zeta_{ij}=1$. We also set $\sum_{j=1}^{0} \zeta_{ij}=0$ for convenience. The values of these simplex parameters are the normalized distances between consecutive stages, e.g. $\zeta_{i2}$ is the distance between stages $j=2$ (moderate dementia) and $j=1$ (mild dementia) for symptom $i$.

We see multiple advantages to this approach over, say, treating stage as a linear term (coding MCI as 0, mild dementia as 1, etc.) or as a categorical term. First, it does not assume equidistance between stages (e.g. if we see a frequency increase of 5% from MCI to mild dementia, we do not assume the same increase from mild to moderate dementia). Second, it gives us parameter and uncertainty estimates that are easy to interpret.

The intercept term $\alpha$ gives us the log-odds estimate of profiles in the reference stage ($j=0$: MCI) selecting the reference symptom ($i=0$: memory of recent events). Every other regression coefficient is interpreted as a change in log-odds relative to those reference categories. $\beta_{i}$ is the change in log-odds for symptom $i$ in the reference stage (MCI). $\gamma_{0}$ and $\gamma_{i}$ are our parameters of interest in this analysis: the former is the average change in log-odds between adjacent stages *for the reference symptom*, and the sum of the two are the average change in log-odds between adjacent stages *for symptom* $i$.

The R package *brms* [80,81] was used to fit the model via Stan, a probabilistic programming language that allows Bayesian inference through Markov chain Monte Carlo (MCMC) methods [82]. We used the following prior distributions for the parameters:

$$\begin{matrix} \alpha& \sim\text{Normal}(-2,2) \\ \beta_{i},\gamma_{0},\gamma_{i} & \sim\text{Normal}(0,2) \\ \zeta_{ij} & \sim\text{Dirichlet}(1,1,1) \end{matrix}$$

The prior on $\alpha$ is wide and shifted to negative log-odds values so that most of the probability mass is below 50% on the frequency scale, and the priors on $\beta_{i}$, $\gamma_{0}$ and $\gamma_{i}$ are wide and centered so to be flexible to changes in either direction. The prior on the simplex parameters is the multivariate Dirichlet distribution which, for a uniform parameter vector of $(1,1,1)$ assumes all differences between adjacent stages to be the same on average but with high uncertainty to allow for all other possible monotonic trends [46]. These priors are considered “weakly informative” so to not bias the inference. The results obtained here would be similar to maximum likelihood inferences, but we are unaware of this sort of monotonic predictor modeling with a frequentist framework implemented in R.

We ran four MCMC chains for 2000 iterations, 500 of which were warmup/adaptation iterations. We assessed convergence by inspecting trace plots (which were well-mixed and stable for all parameters) and by examining $\hat{R}$ values (all values $\leq$ 1.002 indicating excellent convergence).

As a caveat, we don’t expect this modeling approach to fit the data better than other approaches. From a quick look at the frequencies in Table S1, it is clear that many symptoms do not exhibit a consistently increasing or decreasing frequency across stages. Rather, by imposing monotonicity in this way, we are investigating (in a principled, robust way) the hypothesis that there are symptoms characteristic of increasing/decreasing dementia severity.

# Supplementary Tables

Table S1: myGoalNav™ Dementia symptom tracking frequency by stage. The first 60 (59 specific and 1 ‘Other Symptom’) were available in both web and mobile versions of myGoalNav™, and so were included in our analyses. The bottom 8 symptoms, however, were added with launch of the mobile version, and so have less data to draw upon (156 app users). These 8 were excluded from analyses.

| Symptom | Total n=3909 | MCI n=917 | Mild dementia n=1596 | Moderate dementia n=514 | Severe dementia n=882 |
| --- | --- | --- | --- | --- | --- |
| Aggression | 9.7% | 2.2% | 6.7% | 8.9% | 23.2% |
| Anxiety and worry | 14.0% | 11.6% | 16.6% | 19.1% | 9.1% |
| Appetite | 7.4% | 0.8% | 8.6% | 13.6% | 8.3% |
| Attention or concentration | 15.9% | 15.4% | 18.7% | 21.2% | 8.3% |
| Balance | 11.4% | 2.6% | 9.2% | 19.6% | 19.6% |
| Bathing | 5.7% | 0.8% | 5.4% | 15.0% | 6.0% |
| Comprehension | 15.7% | 5.6% | 21.6% | 19.5% | 13.2% |
| Decision making | 10.1% | 8.3% | 12.5% | 13.0% | 6.0% |
| Delusions and paranoia | 9.2% | 2.0% | 8.9% | 14.2% | 14.5% |
| Disorientation to place | 6.4% | 2.8% | 6.9% | 8.0% | 8.5% |
| Disorientation to time | 9.1% | 2.0% | 10.0% | 16.5% | 10.5% |
| Dressing | 5.3% | 0.7% | 4.5% | 15.4% | 5.9% |
| Driving | 6.7% | 2.4% | 9.1% | 9.9% | 5.1% |
| Eating | 7.7% | 1.2% | 5.4% | 12.3% | 16.0% |
| Financial management | 9.7% | 2.9% | 13.9% | 18.7% | 4.0% |
| Following instructions | 8.9% | 5.7% | 11.7% | 12.3% | 5.0% |
| Hallucinations | 5.0% | 1.0% | 3.6% | 7.6% | 10.2% |
| Hobbies or games | 8.4% | 1.4% | 11.7% | 14.8% | 6.2% |
| Household chores | 7.4% | 2.4% | 10.0% | 13.8% | 4.2% |
| Inappropriate behavior | 1.9% | 0.5% | 1.1% | 2.7% | 4.4% |
| Inappropriate language | 5.4% | 4.9% | 5.5% | 8.0% | 4.3% |
| Incontinence | 11.9% | 1.5% | 8.5% | 22.2% | 23.1% |
| Independence | 7.8% | 3.5% | 8.6% | 16.5% | 5.7% |
| Insensitivity | 11.6% | 9.7% | 12.5% | 16.5% | 9.0% |
| Insight | 3.5% | 1.5% | 5.0% | 5.4% | 1.5% |
| Interacting with others | 5.7% | 3.5% | 6.6% | 9.1% | 4.4% |
| Interest or initiative | 14.3% | 10.0% | 21.9% | 14.6% | 4.9% |
| Irritability or frustration | 21.1% | 12.0% | 31.3% | 21.0% | 12.1% |
| Judgment | 8.6% | 5.3% | 9.8% | 15.2% | 6.2% |
| Language difficulty | 16.5% | 15.6% | 19.7% | 11.5% | 14.4% |
| Looking after grandchildren | 2.0% | 0.7% | 2.4% | 4.7% | 1.1% |
| Low mood | 12.2% | 4.3% | 14.8% | 11.9% | 15.8% |
| Low self-esteem | 2.5% | 1.3% | 2.2% | 3.1% | 3.9% |
| Meal preparation or cooking | 9.9% | 2.9% | 15.0% | 15.8% | 4.3% |
| Memory for names and faces | 10.6% | 3.6% | 13.1% | 17.9% | 9.1% |
| Memory of past events | 8.1% | 4.1% | 9.3% | 13.2% | 6.9% |
| Memory of recent events | 29.9% | 33.4% | 36.7% | 37.0% | 9.8% |
| Misplacing or losing objects | 18.6% | 14.5% | 27.1% | 16.9% | 8.5% |
| Mobility | 9.6% | 1.7% | 5.5% | 17.5% | 20.7% |
| Obsessive behavior | 4.2% | 2.4% | 4.6% | 5.6% | 4.6% |
| Operating gadgets or appliances | 6.2% | 1.4% | 8.0% | 13.4% | 3.6% |
| Other symptom | 6.5% | 3.4% | 6.6% | 8.6% | 8.2% |
| Personal care or hygiene | 10.5% | 1.9% | 7.4% | 29.2% | 14.4% |
| Personality changes | 7.7% | 6.0% | 8.8% | 12.6% | 4.8% |
| Physical complaints | 10.2% | 2.2% | 11.7% | 16.9% | 11.8% |
| Reading | 4.7% | 2.2% | 6.2% | 6.8% | 3.2% |
| Repetitive behavior | 5.0% | 1.4% | 3.9% | 8.8% | 8.3% |
| Repetitive questions or stories | 25.8% | 22.8% | 34.8% | 26.5% | 12.2% |
| Restlessness | 4.1% | 0.8% | 2.8% | 5.3% | 9.4% |
| Self-awareness | 2.9% | 2.0% | 3.4% | 6.0% | 1.2% |
| Sensory input | 3.7% | 1.5% | 4.2% | 6.6% | 3.3% |
| Shopping | 4.0% | 1.0% | 5.3% | 9.1% | 1.8% |
| Sleep disturbances | 16.8% | 6.7% | 15.5% | 25.5% | 24.4% |
| Social interaction or withdrawal | 13.7% | 12.4% | 14.9% | 22.4% | 7.8% |
| Spirituality and religion | 1.4% | 0.7% | 1.3% | 3.3% | 1.2% |
| Telephone use | 9.1% | 3.4% | 12.8% | 16.0% | 4.3% |
| Travel | 4.2% | 1.5% | 6.0% | 7.2% | 2.0% |
| Unsafe actions | 4.6% | 2.4% | 4.3% | 10.3% | 4.1% |
| Wandering | 4.4% | 1.3% | 2.0% | 6.6% | 10.7% |
| Writing | 4.1% | 1.6% | 4.9% | 5.4% | 4.4% |

| Symptom | Total n=156 | MCI n=33 | Mild n=63 | Moderate n=20 | Severe n=40 |
| --- | --- | --- | --- | --- | --- |
| Aids/Support devices | 1.9% | 0.0% | 3.2% | 0.0% | 2.5% |
| Conversations | 8.3% | 3.0% | 9.5% | 10.0% | 10.0% |
| Emotional Upset | 3.8% | 3.0% | 6.3% | 0.0% | 2.5% |
| General Memory | 5.1% | 0.0% | 7.9% | 10.0% | 2.5% |
| Relationships | 5.8% | 3.0% | 7.9% | 10.0% | 2.5% |
| Resisting/Refusing | 3.2% | 0.0% | 3.2% | 5.0% | 5.0% |
| Shadowing | 1.3% | 0.0% | 1.6% | 5.0% | 0.0% |
| Thinking & Planning | 2.6% | 3.0% | 1.6% | 10.0% | 0.0% |

Table S2: myGoalNav™ Dementia symptom potency estimates [95% CIs] by stage.

| Symptom | Total n=2874 | MCI n=630 | Mild dementia n=1210 | Moderate dementia n=408 | Severe dementia n=626 |
| --- | --- | --- | --- | --- | --- |
| Aggression | 0.45 [0.43-0.47] | 0.36 [0.30-0.44] | 0.44 [0.41-0.47] | 0.46 [0.41-0.51] | 0.46 [0.43-0.49] |
| Anxiety and worry | 0.53 [0.52-0.55] | 0.61 [0.57-0.65] | 0.51 [0.49-0.53] | 0.57 [0.53-0.60] | 0.49 [0.45-0.53] |
| Appetite | 0.57 [0.55-0.59] | 0.47 [0.35-0.59] | 0.64 [0.61-0.67] | 0.45 [0.42-0.49] | 0.62 [0.58-0.66] |
| Attention or concentration | 0.50 [0.49-0.51] | 0.53 [0.49-0.57] | 0.49 [0.47-0.50] | 0.49 [0.46-0.52] | 0.54 [0.50-0.58] |
| Balance | 0.53 [0.51-0.55] | 0.53 [0.46-0.60] | 0.54 [0.51-0.57] | 0.51 [0.47-0.54] | 0.55 [0.51-0.58] |
| Bathing | 0.52 [0.50-0.54] | 0.68 [0.47-0.84] | 0.54 [0.51-0.58] | 0.49 [0.46-0.53] | 0.52 [0.47-0.56] |
| Comprehension | 0.47 [0.46-0.49] | 0.51 [0.46-0.57] | 0.47 [0.45-0.49] | 0.43 [0.40-0.47] | 0.53 [0.49-0.56] |
| Decision making | 0.49 [0.47-0.51] | 0.50 [0.44-0.55] | 0.46 [0.44-0.48] | 0.50 [0.46-0.53] | 0.60 [0.55-0.64] |
| Delusions and paranoia | 0.52 [0.51-0.54] | 0.55 [0.47-0.63] | 0.54 [0.51-0.57] | 0.49 [0.45-0.53] | 0.53 [0.49-0.56] |
| Disorientation to place | 0.56 [0.54-0.58] | 0.57 [0.46-0.68] | 0.59 [0.56-0.62] | 0.50 [0.45-0.55] | 0.55 [0.51-0.59] |
| Disorientation to time | 0.56 [0.54-0.57] | 0.56 [0.46-0.66] | 0.55 [0.53-0.58] | 0.53 [0.50-0.56] | 0.59 [0.56-0.63] |
| Dressing | 0.58 [0.56-0.60] | 0.52 [0.39-0.64] | 0.51 [0.47-0.55] | 0.62 [0.59-0.65] | 0.61 [0.56-0.65] |
| Driving | 0.62 [0.60-0.64] | 0.74 [0.66-0.81] | 0.59 [0.57-0.62] | 0.67 [0.63-0.71] | 0.60 [0.55-0.66] |
| Eating | 0.51 [0.49-0.53] | 0.79 [0.68-0.87] | 0.54 [0.50-0.58] | 0.48 [0.45-0.52] | 0.48 [0.44-0.51] |
| Financial management | 0.60 [0.58-0.62] | 0.60 [0.52-0.68] | 0.61 [0.59-0.63] | 0.57 [0.54-0.60] | 0.67 [0.61-0.73] |
| Following instructions | 0.52 [0.50-0.54] | 0.55 [0.49-0.61] | 0.52 [0.49-0.54] | 0.53 [0.49-0.57] | 0.50 [0.45-0.55] |
| Hallucinations | 0.51 [0.49-0.54] | 0.50 [0.37-0.63] | 0.54 [0.50-0.59] | 0.55 [0.49-0.60] | 0.48 [0.44-0.52] |
| Hobbies or games | 0.71 [0.70-0.73] | 0.85 [0.75-0.92] | 0.71 [0.69-0.74] | 0.71 [0.68-0.74] | 0.68 [0.63-0.73] |
| Household chores | 0.66 [0.64-0.68] | 0.65 [0.57-0.72] | 0.66 [0.63-0.68] | 0.67 [0.64-0.70] | 0.66 [0.61-0.71] |
| Inappropriate behavior | 0.55 [0.50-0.59] | 0.82 [0.65-0.92] | 0.71 [0.60-0.80] | 0.46 [0.38-0.54] | 0.49 [0.41-0.56] |
| Inappropriate language | 0.56 [0.54-0.59] | 0.68 [0.60-0.75] | 0.56 [0.52-0.59] | 0.52 [0.47-0.57] | 0.56 [0.51-0.62] |
| Incontinence | 0.52 [0.50-0.53] | 0.81 [0.64-0.91] | 0.58 [0.55-0.61] | 0.47 [0.44-0.50] | 0.50 [0.47-0.53] |
| Independence | 0.56 [0.54-0.58] | 0.56 [0.49-0.64] | 0.55 [0.53-0.58] | 0.55 [0.52-0.59] | 0.60 [0.56-0.64] |
| Insensitivity | 0.53 [0.52-0.55] | 0.60 [0.55-0.65] | 0.53 [0.51-0.55] | 0.52 [0.48-0.56] | 0.51 [0.48-0.55] |
| Insight | 0.61 [0.59-0.64] | 0.58 [0.49-0.67] | 0.61 [0.58-0.64] | 0.59 [0.53-0.64] | 0.78 [0.69-0.85] |
| Interacting with others | 0.64 [0.62-0.66] | 0.70 [0.62-0.76] | 0.64 [0.61-0.67] | 0.67 [0.62-0.71] | 0.59 [0.54-0.65] |
| Interest or initiative | 0.63 [0.61-0.64] | 0.62 [0.58-0.67] | 0.65 [0.63-0.67] | 0.60 [0.57-0.64] | 0.50 [0.44-0.56] |
| Irritability or frustration | 0.51 [0.50-0.52] | 0.51 [0.48-0.55] | 0.51 [0.50-0.53] | 0.52 [0.49-0.55] | 0.50 [0.46-0.53] |
| Judgment | 0.52 [0.50-0.54] | 0.50 [0.45-0.56] | 0.49 [0.46-0.52] | 0.53 [0.49-0.57] | 0.59 [0.54-0.63] |
| Language difficulty | 0.48 [0.46-0.49] | 0.57 [0.53-0.61] | 0.43 [0.41-0.45] | 0.51 [0.47-0.55] | 0.50 [0.47-0.53] |
| Looking after grandchildren | 0.75 [0.72-0.78] | 0.89 [0.78-0.95] | 0.72 [0.67-0.76] | 0.78 [0.72-0.83] | 0.74 [0.64-0.82] |
| Low mood | 0.51 [0.49-0.52] | 0.47 [0.41-0.52] | 0.53 [0.51-0.56] | 0.42 [0.38-0.46] | 0.52 [0.49-0.55] |
| Low self-esteem | 0.53 [0.49-0.56] | 0.45 [0.36-0.55] | 0.51 [0.45-0.56] | 0.57 [0.49-0.65] | 0.56 [0.49-0.62] |
| Meal preparation or cooking | 0.60 [0.59-0.62] | 0.66 [0.59-0.73] | 0.60 [0.57-0.62] | 0.59 [0.56-0.62] | 0.68 [0.62-0.74] |
| Memory for names and faces | 0.57 [0.55-0.59] | 0.60 [0.52-0.67] | 0.52 [0.50-0.55] | 0.59 [0.56-0.63] | 0.66 [0.62-0.70] |
| Memory of past events | 0.57 [0.55-0.59] | 0.61 [0.55-0.67] | 0.56 [0.53-0.59] | 0.55 [0.50-0.59] | 0.61 [0.57-0.65] |
| Memory of recent events | 0.44 [0.43-0.45] | 0.53 [0.50-0.57] | 0.42 [0.41-0.44] | 0.41 [0.38-0.44] | 0.51 [0.47-0.55] |
| Misplacing or losing objects | 0.52 [0.51-0.54] | 0.66 [0.61-0.70] | 0.50 [0.48-0.51] | 0.50 [0.47-0.53] | 0.59 [0.56-0.63] |
| Mobility | 0.50 [0.49-0.52] | 0.64 [0.55-0.73] | 0.59 [0.56-0.63] | 0.45 [0.42-0.48] | 0.47 [0.45-0.50] |
| Obsessive behavior | 0.52 [0.49-0.54] | 0.47 [0.39-0.54] | 0.54 [0.51-0.58] | 0.47 [0.41-0.53] | 0.51 [0.46-0.57] |
| Operating gadgets or appliances | 0.66 [0.64-0.68] | 0.48 [0.33-0.62] | 0.65 [0.62-0.68] | 0.67 [0.64-0.71] | 0.68 [0.62-0.74] |
| Other symptom | 0.62 [0.60-0.64] | 0.70 [0.63-0.76] | 0.64 [0.61-0.67] | 0.56 [0.51-0.61] | 0.59 [0.55-0.64] |
| Personal care or hygiene | 0.53 [0.51-0.55] | 0.51 [0.42-0.59] | 0.54 [0.50-0.57] | 0.50 [0.47-0.53] | 0.56 [0.53-0.60] |
| Personality changes | 0.51 [0.49-0.53] | 0.51 [0.45-0.57] | 0.49 [0.46-0.52] | 0.54 [0.50-0.58] | 0.56 [0.50-0.62] |
| Physical complaints | 0.61 [0.59-0.63] | 0.63 [0.54-0.71] | 0.62 [0.59-0.64] | 0.56 [0.53-0.60] | 0.65 [0.61-0.68] |
| Reading | 0.64 [0.62-0.66] | 0.66 [0.56-0.75] | 0.65 [0.62-0.68] | 0.59 [0.54-0.64] | 0.67 [0.61-0.73] |
| Repetitive behavior | 0.55 [0.52-0.57] | 0.43 [0.33-0.54] | 0.53 [0.48-0.57] | 0.57 [0.52-0.62] | 0.59 [0.54-0.63] |
| Repetitive questions or stories | 0.42 [0.41-0.43] | 0.48 [0.44-0.52] | 0.39 [0.38-0.41] | 0.43 [0.40-0.46] | 0.48 [0.45-0.52] |
| Restlessness | 0.58 [0.55-0.60] | 0.50 [0.39-0.61] | 0.58 [0.53-0.62] | 0.58 [0.52-0.64] | 0.59 [0.54-0.63] |
| Self-awareness | 0.56 [0.53-0.59] | 0.44 [0.36-0.52] | 0.55 [0.51-0.59] | 0.58 [0.53-0.63] | 0.68 [0.60-0.75] |
| Sensory input | 0.65 [0.62-0.67] | 0.53 [0.43-0.62] | 0.64 [0.61-0.68] | 0.66 [0.61-0.71] | 0.68 [0.63-0.74] |
| Shopping | 0.67 [0.64-0.69] | 0.72 [0.61-0.81] | 0.66 [0.63-0.69] | 0.68 [0.64-0.72] | 0.60 [0.50-0.69] |
| Sleep disturbances | 0.55 [0.54-0.57] | 0.61 [0.56-0.66] | 0.60 [0.58-0.62] | 0.51 [0.48-0.54] | 0.50 [0.48-0.53] |
| Social interaction or withdrawal | 0.65 [0.63-0.66] | 0.67 [0.64-0.71] | 0.67 [0.65-0.69] | 0.60 [0.57-0.63] | 0.63 [0.59-0.67] |
| Spirituality and religion | 0.67 [0.63-0.71] | 0.54 [0.42-0.65] | 0.69 [0.63-0.75] | 0.71 [0.63-0.77] | 0.66 [0.57-0.74] |
| Telephone use | 0.67 [0.65-0.68] | 0.74 [0.65-0.81] | 0.68 [0.65-0.70] | 0.64 [0.61-0.67] | 0.66 [0.61-0.71] |
| Travel | 0.80 [0.78-0.82] | 0.82 [0.74-0.88] | 0.80 [0.77-0.82] | 0.82 [0.79-0.86] | 0.74 [0.66-0.80] |
| Unsafe actions | 0.53 [0.50-0.55] | 0.63 [0.53-0.72] | 0.61 [0.57-0.65] | 0.46 [0.41-0.51] | 0.43 [0.38-0.49] |
| Wandering | 0.48 [0.45-0.50] | 0.69 [0.60-0.77] | 0.46 [0.39-0.53] | 0.45 [0.40-0.51] | 0.46 [0.42-0.50] |
| Writing | 0.65 [0.63-0.68] | 0.74 [0.64-0.82] | 0.63 [0.60-0.67] | 0.63 [0.57-0.69] | 0.69 [0.64-0.74] |

# Supplementary Figures


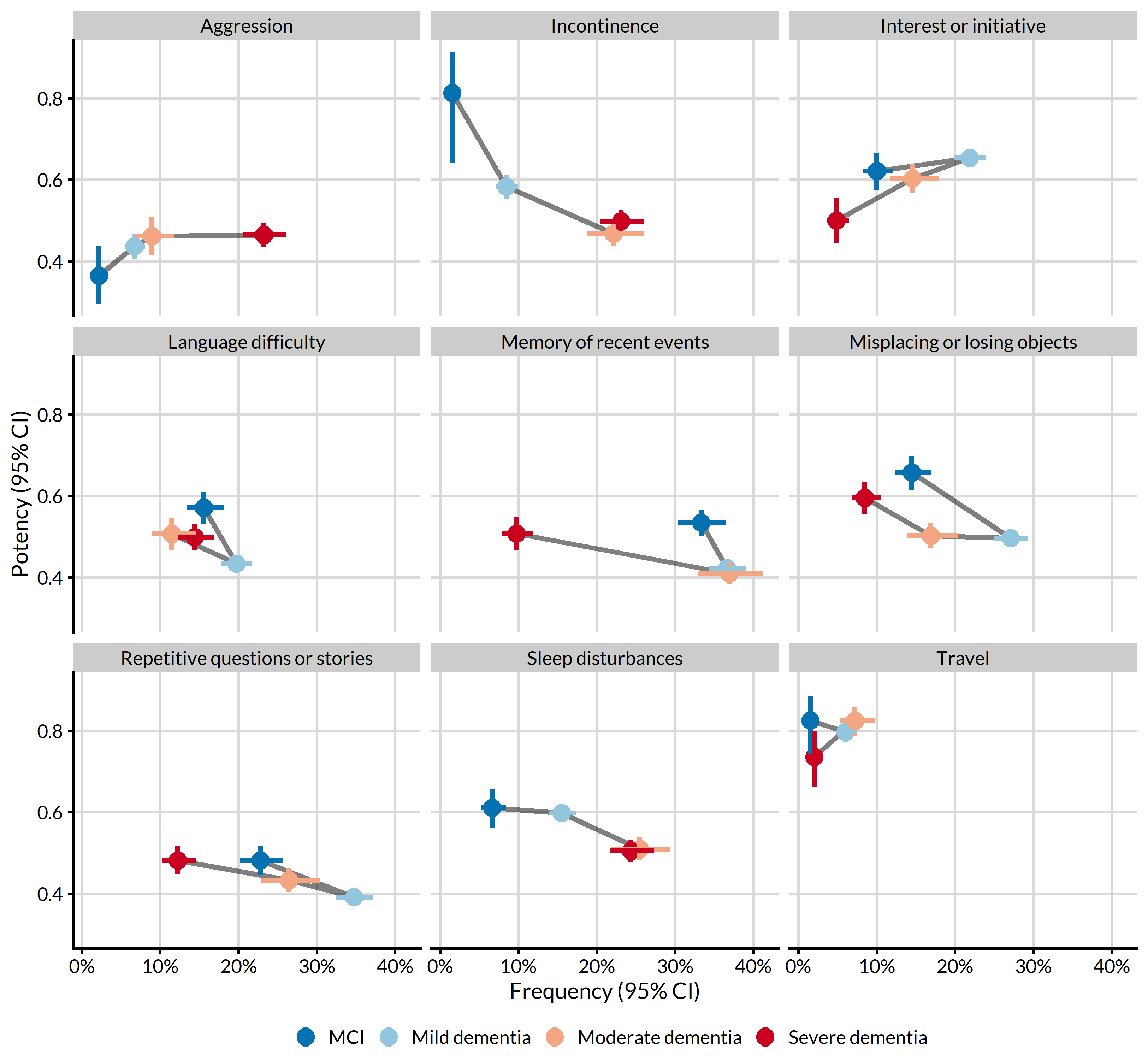


Figure S1: The relationship between frequency and potency at each stage for selected symptoms. Bars indicatse 95% confidence intervals. The dark grey lines connect the stages in order of severity.

# References

46. Bürkner P-C, Charpentier E. Modelling monotonic effects of ordinal predictors in Bayesian regression models. British Journal of Mathematical and Statistical Psychology 2020 Jan;bmsp.12195. PMID:[31943157](https://www.ncbi.nlm.nih.gov/pubmed/31943157)

80. Bürkner P-C. brms : An R Package for Bayesian Multilevel Models Using Stan. Journal of Statistical Software 2017;80(1). [doi: [10.18637/jss.v080.i01](https://doi.org/10.18637/jss.v080.i01)]

81. Bürkner P-C. Advanced Bayesian Multilevel Modeling with the R Package brms. The R Journal 2018;10(1):395. [doi: [10.32614/RJ-2018-017](https://doi.org/10.32614/RJ-2018-017)]

82. Carpenter B, Gelman A, Hoffman MD, Lee D, Goodrich B, Betancourt M, Brubaker M, Guo J, Li P, Riddell A. Stan : A Probabilistic Programming Language. Journal of Statistical Software 2017;76(1). [doi: [10.18637/jss.v076.i01](https://doi.org/10.18637/jss.v076.i01)]
